# Mitochondrial Genome Engineering: The Revolution May Not Be CRISPR-Ized

**DOI:** 10.1016/j.tig.2017.11.001

**Published:** 2018-02

**Authors:** Payam A. Gammage, Carlos T. Moraes, Michal Minczuk

**Affiliations:** 1 Medical Research Council (MRC) Mitochondrial Biology Unit, University of Cambridge, Cambridge, UK; 2 Miller School of Medicine, University of Miami, Miami, FL, USA

**Keywords:** mitochondria, mtDNA, CRISPR/Cas9, mtZFN, mitoTALEN, RNA import

## Abstract

In recent years mitochondrial DNA (mtDNA) has transitioned to greater prominence across diverse areas of biology and medicine. The recognition of mitochondria as a major biochemical hub, contributions of mitochondrial dysfunction to various diseases, and several high-profile attempts to prevent hereditary mtDNA disease through mitochondrial replacement therapy have roused interest in the organellar genome. Subsequently, attempts to manipulate mtDNA have been galvanized, although with few robust advances and much controversy. Re-engineered protein-only nucleases such as mtZFN and mitoTALEN function effectively in mammalian mitochondria, although efficient delivery of nucleic acids into the organelle remains elusive. Such an achievement, in concert with a mitochondria-adapted CRISPR/Cas9 platform, could prompt a revolution in mitochondrial genome engineering and biological understanding. However, the existence of an endogenous mechanism for nucleic acid import into mammalian mitochondria, a prerequisite for mitochondrial CRISPR/Cas9 gene editing, remains controversial.

## Trends

Engineering of mammalian mtDNA has been hampered by an inability to import nucleic acids into mitochondria.

A limited toolkit exists for manipulation of mammalian mtDNA, relying on protein-only nucleolysis and heteroplasmy-shifting approaches.

Although present in lower metazoans, the weight of evidence against an efficient endogenous RNA import mechanism in mammalian mitochondria is considerable.

Controversially, the application of CRISPR/Cas9 for manipulation of mammalian mtDNA in human cells has been reported.

Biological understanding of complex organisms in the modern era relies heavily on reverse genetics. As an area of interest for many, a robust method for directed genetic manipulation of mammalian mitochondria has been sought for several decades. More recently, efforts to this end have largely focused on the search for treatments of mitochondrial disease. Incurable and largely intractable, mitochondrial diseases caused by mutation of the mitochondrial genome affect approximately one in 5000 and represent a substantial disease burden [1]. The dawn of the genome-editing era augurs well for both basic and clinical mitochondrial research, and the CRISPR/Cas9 revolution in particular seems to bring a paradigm shift within our grasp. However, fundamental questions regarding the capacity of mammalian mitochondria to import the **guide RNA** (gRNA; see Glossary) molecules needed for a viable CRISPR/Cas9 system cast doubt upon such an enterprise. Over recent years evidence against the notion of endogenous import of nucleus-encoded RNA into mammalian mitochondria has accrued. In this article we discuss the mitochondrial genetic system, evidence for and against endogenous RNA import into, and proposed functions within, mammalian mitochondria, and recent efforts towards genetic manipulation of mitochondria, including the controversial report of a mitochondrial CRISPR/Cas9 system.

## Molecular Biology of Mammalian Mitochondria

From the initial alphaproteobacterial engulfment, that formed the first eukaryote through endosymbiosis, to the present-day organelle residing in mammalian cells, the relationship between mitochondria and their hosts has evolved substantially. Where once mitochondria-like symbionts were advantageous principally for their capacity to harness redox chemistries, the role of mitochondria in diverged eukaryotes, such as mammals, is much more intricately embedded in essential organismal function. Facilitation of these functions relies upon an electrochemical disequilibrium potential across the **inner mitochondrial membrane** (IMM) that is generated through proton pumping by respiratory chain complexes I, III, and IV. Taken together, the respiratory chain and ATP synthase consist of ∼90 protein subunits, forming IMM-bound protein complexes. The vast majority of these proteins are encoded in and expressed from the nuclear genome; however, a subset is encoded within a spatially and heritably separate genome – the mitochondrial genome.

Mammalian mitochondrial DNA (mtDNA) is a multi-copy, circular, double-stranded DNA molecule encoding 13 essential membrane-bound polypeptide subunits of the respiratory chain complexes I, III, IV, and ATP synthase, 22 tRNAs, and two ribosomal RNAs (rRNAs). At ∼16.5 kb, mammalian mtDNAs are relatively small and genetically compact, containing very little non-coding sequence and two overlapping genes [2]. The mitochondrial genome is packaged into individual nucleoids that consist principally of the mitochondrial transcription factor A (TFAM) 3, 4, but likely also contain other factors 5, 6, 7, and these nucleoids are tightly associated with the IMM within the matrix. The mechanism by which mtDNA is replicated has, over the years, been no small matter of debate 8, 9, 10, with recent data pointing towards the originally proposed strand-displacement mechanism 11, 12, 13. Transcription of mtDNA occurs from the **heavy-strand** promoter (HSP) and the **light-strand** promoter (LSP), resulting in polycistronic transcripts that undergo substantial processing to yield the mature mRNA, tRNA, and rRNA molecules that are required for translation by mitochondrial ribosomes (mitoribosomes) 14, 15. A diverse array of DNA repair pathways exist in mammalian mitochondria 16, 17, with the notable absence of efficient DNA double-strand break (DSB) repair [18], and either inefficient or absent homologous recombination (HR) [19]. The mitochondrial genome is, in mammals, strictly maternally inherited, demonstrating a more stochastic mode of transmission than Mendelian genetics as a consequence of the mtDNA **bottleneck** [20]. Diseases arising from mutations in mtDNA most often present in a **heteroplasmic** state, where a substantial proportion of mtDNA molecules bear a pathogenic mutation that is partially rescued by the presence of wild-type molecules in the same cell [21].

## A Role for Endogenous RNA Import in Mammalian Mitochondria

**Figure 1:**
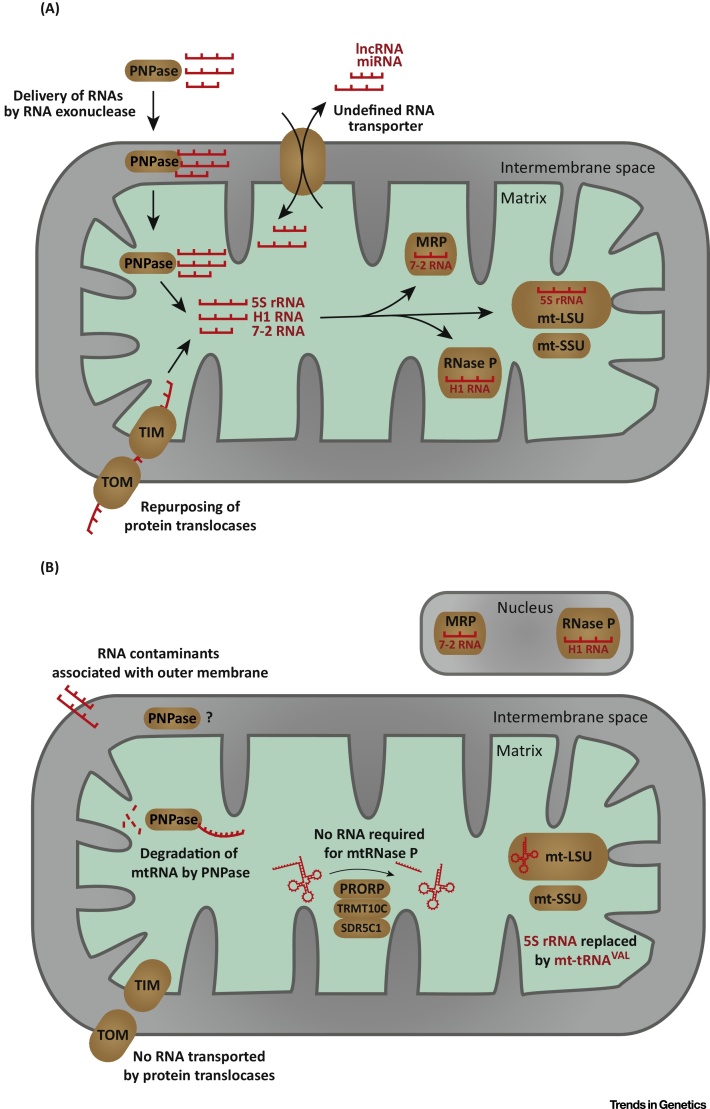
Overview of Putative RNA Import into Mitochondria. (A) An overview of historically proposed mechanisms and functional roles of endogenous RNAs imported into mammalian mitochondria. Nucleus-encoded RNA is suggested to enter the mitochondrial matrix in complex with polynucleotide phosphorylase (PNPase), via the mitochondrial protein translocase of outer membrane (TOM) and translocase of inner membrane (TIM), as well as by other undescribed and undefined mechanisms of transport. Endogenous RNA species with previously proposed functional roles in mammalian mitochondria are H1 RNA (RNase P), 7-2 RNA (RNase MRP), and 5S rRNA (mt-LSU). (B) A revised overview of the proposed mechanisms and functional roles of endogenous RNAs imported into mammalian mitochondria, modified to reflect findings from recent papers concerning (i) the function of PNPase as a key constituent of the mtRNA degradasome 54, 61, (ii) replacement of 5S rRNA in mt-LSU of the mitoribosome by mitochondrial tRNA 49, 98, (iii) discovery of protein-only RNase P (PRORP) in mammalian mitochondria [31], and (iv) reattribution of RNase MRP activity to the nucleolus, similarly to nuclear RNase P 37, 38. Many of the RNAs ‘detected’ and ascribed mitochondrial functions are predicted to be false positives as a result of contamination of mitochondrial preparations with cytosolic RNAs or with RNAs associated with the outer membrane, such as mRNAs encoding mitochondrial proteins that undergo co-translational import into mitochondria 65, 66, 99. A question mark indicates unconfirmed function and/or localization of PNPase. Abbreviations: mt-LSU, mitoribosome large subunit; mtRNA, mitochondrial RNA; mt-SSU, mitoribosome small subunit.

It is well-established that 11 protein-coding mRNAs, encoding 13 polypeptides of respiratory chain complexes and ATP synthase, are transcribed from the mitochondrial genome and translated by mitoribosomes. In placental mammals a full complement of 22 functional tRNA species capable of recognizing 60 sense codons, and two rRNAs that are required for translation by mitoribosomes, are also encoded in mtDNA. Considering the substantial structural differences between mitochondrial and cytosolic tRNAs, the divergence and incompatibility of codon usage between mitochondrial and nuclear mRNAs, the lack of unassigned codons in mitochondrial open reading frames (ORFs) [22], and that all other mitochondrial proteins are encoded and expressed from the nuclear genome, any mRNA-decoding function for RNA imported into mitochondria is not immediately apparent. However, various other roles for endogenous, nuclear-encoded RNAs imported into mitochondria have been debated (Figure 1A,B).

An unusual, bacteria-like feature of mammalian mitochondrial gene expression is the near-unit length polycistronic transcripts produced through transcription of mtDNA. Within the polycistrons, most gene products are punctuated by one or more tRNAs, which require endonucleolytic processing at both 5′ and 3′ ends to release individual transcripts, a concept termed ‘tRNA punctuation’ [23]. Essential to this process is **mitochondrial RNase P** (mtRNase P). Both nuclear and mitochondrial RNases P liberate the 5′ ends of immature tRNA transcripts through structure-guided endonucleolytic processing. RNase P is an ancient enzyme, initially identified in bacteria, followed by eukaryotic nuclei and yeast mitochondria 24, 25, 26. Among several protein subunits, the nuclear (n)RNase P holoenzyme contains a single RNA subunit (**H1 RNA**) that is necessary for catalytic function 27, 28. It was shown that mtDNA of some fungi and protists encode RNase P RNA, therefore it was assumed, and later controversially reported, that mammalian mtRNase P would require a catalytic RNA subunit to function 29, 30. However, it has since emerged that human mtRNase P, a product of convergent evolution, bears no relation to nRNase P [31]. Human mtRNase P consists of three protein subunits: a mitochondrially targeted tRNA m^1^R methyltransferase, TRMT10C (MRPP1), a member of the short-chain dehydrogenase/reductase (SDR) family, SDR5C1 (HSD17B10, MRPP2), and a protein with homology to **PiIT N terminus** (PIN) domain-like metallonucleases, PRORP (MRPP3) 31, 32. Importantly, the human mtRNase P was shown specifically not to contain any *trans*-acting RNA. This paradigm-shifting subclass of **proteinaceous RNase P** (PRORP) has since been identified in most eukaryal lineages (33, 34 for an in-depth review on the discovery and evolution of PRORPs).

A further controversy concerning mitochondrial RNA processing by imported endogenous RNAs concerns the nRNase P-related endonuclease, the mitochondrial RNA processing ribonuclease (**RNase MRP**). Similarly to nRNase P, RNase MRP possesses a RNA subunit (termed **7-2 RNA** in the early literature) and several protein components, most of which are shared with nRNase P [35]. RNase MRP was first described as a ribonucleoprotein complex present in mitochondria that is involved in the formation of a RNA primer during initiation of mammalian mtDNA replication [36]. However, subsequent studies have provided compelling evidence against a mitochondrial localization of 7-2 RNA in mammalian cells, arguing that RNase MRP, like nRNase P, is found mainly in the nucleolus 37, 38 where it plays an essential role in pre-ribosomal RNA processing [39]. In addition, *in vitro* reconstitution experiments have suggested an RNase MRP-independent mechanism for primer processing in mtDNA replication, where the 3'-end of the RNA primer is generated by site-specific termination of transcription owing to G-quadruplex formation in nascent RNA, rather than cleavage by RNase MRP [40]. These findings point away from the requirement for non-mtDNA transcribed RNA to be present in mitochondria for RNA processing, suggesting that endogenous RNA import into mammalian mitochondria is not required for normal cellular functions.

Another area of debate concerning mitochondrial import of endogenous RNA in mammals focuses on the RNA content of mitoribosomes, specifically the existence of a minor structural rRNA species analogous to the **5S rRNA** that is found in ribosomes from other cellular compartments and organisms. Several groups have argued in favor of 5S rRNA being present in mammalian mitochondria 41, 42, 43, 44. Key determinants of efficient 5S rRNA import are suggested to include specific RNA structural folds and protein cofactors 45, 46, and incorporation of 5S rRNA into the mitoribosome through interactions with proposed mitoribosomal protein MRPL18 has been described [47]. However, the notion of 5S rRNA incorporation within the mitoribosome has been categorically disregarded since publication of high-resolution structures of porcine and human mitoribosomes demonstrating that a mtDNA-encoded tRNA (mt-tRNA), either mt-tRNA^Phe^ or mt-tRNA^Val^, is embedded in the large subunit of the mammalian mitoribosome (mt-LSU), to the exclusion of any 5S rRNA molecule, and that would require substantial remodeling of the mitoribosomal central protuberance to accommodate 5S rRNA 48, 49, 50, 51. Further, it has been reported that a homoplasmic disease-causative point mutation in mt-tRNA^Val^ leads to destabilization of this tRNA and a switch in the structural RNA content of mt-LSU from mt-tRNA^Val^ to mt-tRNA^Phe^ 52, 53. These data raise questions regarding a physiological role for imported RNAs in mammalian mitoribosomes.

A factor suggested to directly facilitate endogenous RNA import into mammalian mitochondria is a component of the mitochondrial RNA degradation machinery, **polynucleotide phosphorylase** (PNPase). PNPase is a homotrimeric 3′–5′ exoribonuclease which, together with mitochondrial RNA-specific helicase, hSUV3, forms the RNA degradasome in the mitochondrial matrix 54, 55. However, an alternative function and localization of PNPase has been proposed. Detection of PNPase in the mitochondrial intermembrane space (IMS), rather than in the matrix, has led to suggestions that it could mediate mitochondrial matrix translocation of 5S rRNA, H1 RNA, 7-2 RNA, and more recently also microRNAs (miRNAs) by an uncharacterized mechanism 56, 57. This was surprising because PNPases, an ancient family of enzymes, had previously been found to reside in the matrix and to be involved in degradation of RNA, rather than in transport 54, 58, 59, 60. Interestingly, pathogenic compound heterozygous mutations in the PNPase gene (*PNPT1*), that were predicted to disrupt the homotrimer and therefore abolish any catalytic or transport function of PNPase, led to an accumulation of aberrantly processed mitochondrial RNA species within mitochondria, in line with the expectation of a role for PNPase in degradation of mitochondrial RNA [61]. Notably, the accumulated RNA intermediates were correctly processed at 5′ tRNA junctions, strongly suggesting that any mitochondrial import of H1 RNA by PNPase is dispensable for function of mitochondrial RNase P, as previously discussed. Given the consensus localization of PNPase in the mitochondrial matrix [100], its well-described role in mitochondrial RNA degradation, the lack of a well-understood RNA import mechanism, and the likely dispensable role of RNAs it is alleged to transport, PNPase-mediated RNA import into mammalian mitochondria is not widely accepted, and requires further exploration and confirmation.

In addition to the research concerning import of endogenous RNAs into mammalian mitochondria, discussed above, there also exists a less well interrogated literature suggesting both import and export of miRNAs [62], long non-coding RNAs (lncRNAs) [63], and tRNAs [64] into and from mammalian mitochondria, which will not be discussed here because we believe this requires validation by independent studies.

It is helpful to underscore the often-contradictory findings reported in the studies discussed above by reference to valuable data from a comprehensive, quantitative analysis of the human mitochondrial transcriptome [65]. In this study, numerous nucleus-encoded RNA species were detected in enriched mitochondrial RNA samples, although upon disruption and removal of the outer mitochondrial membrane these RNAs were almost exclusively found to be less abundant or only fractionally enriched; by contrast, *bona fide* mtDNA-encoded RNA species were enriched by many orders of magnitude. Such outer-membrane contaminants have been long discussed [66], and are a likely source of confusion and controversy within the field.

## Manipulation of the Mitochondrial Genome by Protein-Only Nucleases

Despite innumerable innovative investigations that have yielded a substantial but mosaic literature on the subject, the vast majority of approaches to mitochondrial genome engineering have failed to be either efficient or robust, and progress has been glacial (as recently reviewed [67]). Presently, reliable methods for the transformation of mitochondria exist only for yeasts 68, 69 and green algae [70] by means of **biolistics** in combination with endogenous mtDNA HR, which is highly active in these organisms. As the introduction of exogenous nucleic acids into mammalian mitochondria has been resoundingly unsuccessful thus far, alternative approaches to manipulating mtDNA *in situ* have emerged, relying on recognition and specific elimination of targeted mtDNA molecules present in a heteroplasmic population. Such developments hold significant potential for broad application in the treatment of currently incurable diseases arising from mutation of the mitochondrial genome.

Early attempts to manipulate mtDNA heteroplasmy exploited bacterial restriction endonucleases (REs) directed to mitochondria by means of a N-terminal **mitochondrial targeting sequence** (MTS). Once imported into mitochondria, these REs (mtREs) bind to and cleave a DNA recognition site that is present in only one mtDNA haplotype. Because mammalian mitochondria do not possess any efficient DSB repair pathways, mtDNA molecules bearing DSBs are rapidly degraded, producing a shift in the heteroplasmic ratio 71, 72. This strategy is particularly effective because cells typically maintain a steady mtDNA **copy number**; sudden depletion of this copy number by DSB-mediated degradation results in replication of the remaining, intact mtDNA molecules, repopulating cells with the untargeted mtDNA haplotype. Further work with mtREs demonstrated their mtDNA heteroplasmy-shifting efficacy across multiple tissues in both transgenic and **adeno-associated virus** (AAV)-treated mice 73, 74, 75. Despite their capacity to produce large shifts in mtDNA heteroplasmy, the use of mtREs in mitochondrial genome manipulation is limited by their indisposition to protein engineering, preventing the development of alternative DNA recognition site specificities and generalization of this method to diverse genetic variants. As such, a new class of mitochondrially targeted engineered nucleases has emerged that exploit transcription activator-like effector (TALE) and zinc-finger DNA-binding technology – mitochondrially targeted TALE nucleases (mitoTALENs) [76] and mitochondrially targeted zincfinger-nucleases (mtZFNs) [77].

These platforms exploit developments in engineered nuclease technology for nuclear DNA manipulation 78, 79, repurposed to function in mitochondria. Both mtZFNs and mitoTALENs are localized to mitochondria using broadly interchangeable MTS peptides, although mtZFNs require addition of a nuclear export signal (NES) to overcome intrinsic nuclear localization [80]. Once delivered to mitochondria, both platforms have demonstrated a capacity to induce large shifts in mtDNA heteroplasmy, with concomitant physiological rescue, across a range of cells bearing numerous disease-causative genetic variants 76, 77, 81, 82, 83, 84, 85.

Despite being a relatively new addition to the literature, the principles underlying mtZFNs and mitoTALENs have recently been applied by an independent laboratory to answer questions of basic mitochondrial molecular genetics [13]. It seems likely that these approaches to mitochondrial genome manipulation will be further generalized in both basic and clinical research.

## Import of Exogenous RNA and CRISPR/Cas9 in Mammalian Mitochondria

Over the past two decades a series of reports have suggested that RNA import into mammalian mitochondria could be facilitated by exogenous factors. One report described that addition of a multisubunit complex of approximately 500 kDa, isolated from mitochondria of *Leishmania*, to cultured human cells facilitated mitochondrial import of RNA from the cytosol 86, 87. However, an editorial expression of concern regarding this study was published by the *Proceedings of the National Academy of Sciences* [88]. Two corrections to a subsequent paper describing *Leishmania* complex-mediated RNA import [89] have also been published 90, 91. It therefore remains unclear whether such machinery is effective in mammalian mitochondria.

Other studies have claimed delivery of synthetic RNA to mammalian mitochondria through the use of two domains from yeast cytosolic tRNA^Lys(CUU)^, which was demonstrated to partially localize to yeast mitochondria under stress conditions by the same investigators 92, 93. Import of this molecule into mitochondria is proposed to be contingent on adoption of a non-canonical structure, which produces a novel loop known as the F-arm, in addition to the D-loop domain that is present in both canonical and non-canonical structures, referred to as a D-hairpin. Through incorporation of F-arm or D-hairpin motifs into synthetic RNA molecules, efficient delivery of RNA into mammalian mitochondria has been reported, and mtDNA mutation-specific complementary RNAs were shown to specifically stall mutant mtDNA replication and shift heteroplasmy 94, 95. Further studies utilizing domains identified in H1 RNA [96] or 5S rRNA [44] suggested that these could also function as targeting vectors for mitochondrial import of RNA molecules. However, the lack of any accepted molecular mechanism for RNA import into mammalian mitochondria, and the lack of proliferation of these methods beyond their laboratories of origin, preclude general acceptance of effective engineered RNA import.

Given the controversial nature of mammalian mitochondrial RNA import, the publication of a study from Jo and colleagues, employing CRISPR/Cas9 to successfully manipulate mtDNA, was surprising [97]. In a series of experiments, despite a lack of MTS peptides, the authors demonstrated mitochondrial localization of Cas9 bearing a nuclear localization signal (NLS), and gRNAs specific to mitochondrial sequences appeared to allow specific depletion of targeted portions of mtDNA while untargeted regions were enriched. Interestingly, no modifications were made to the gRNAs, and no problems were reported in delivering gRNAs to mitochondria. A mitochondrially targeted form of Cas9 demonstrated the same specific depletion of a single locus of mtDNA based on gRNA recognition and cleavage, although no mtDNA sequencing data were presented for the reportedly modified regions.

Taken together, or separately, these data are extraordinary. The activity of CRISPR/Cas9 in mitochondria without any additional RNA import sequence implies that gRNAs were spontaneously imported into mitochondria from the cytosol. In addition, the data suggest that Cas9 protein has a previously undescribed, potent tropism for mitochondria because Cas9 bearing a NLS was localized to mitochondria. Further, the observation that targeting a single site in mtDNA for cleavage by CRISPR/Cas9 leads to depletion of only that locus, but not degradation of the entire molecule, is difficult to reconcile with the fact that mtDNA behaves as a unit [18]. At present, the data reported by Jo and colleagues falls well short of providing reasonable evidence that CRISPR/Cas9 technology can be used to edit the mitochondrial genome in mammalian systems.

## Concluding Remarks and Future Perspectives

Manipulation of mtDNA is an objective sought by many, from the laboratory to the bedside. The recent CRISPR/Cas9 revolution has transformed nuclear DNA manipulation, heralding the dawn of a new era in molecular biology and gene therapy. Although, given the many questions that remain regarding effective import of either endogenous or exogenous RNAs into mammalian mitochondria, it seems that the genome of this unusual organelle may be one of few genetic systems beyond the reach of CRISPR/Cas9 (see Outstanding Questions).

Reliable import of nucleic acids into mammalian mitochondria, in concert with molecular tools currently in hand, would significantly advance the state of the art, and a functional CRISPR/Cas9 architecture in mitochondria could be revolutionary. However, given current prospects for efficient import of nucleic acids into mammalian mitochondria, a key prerequisite for genome editing by CRISPR/Cas9, the dream of a mitochondrial CRISPR/Cas9 panacea appears destined for a rude awakening.

Outstanding Questions Can RNA be imported into mammalian mitochondria? If so, what is the mechanism underlying this process?

Given the dispensable/unknown function of RNAs proposed to be imported into mammalian mitochondria, what is the purpose of RNA import in such a system?

How could gRNA be delivered to mammalian mitochondria in a manner compatible with efficient CRISPR/Cas9-mediated gene editing?

## Glossary

**Adeno-associated virus (AAV)**: a widely-used vehicle for gene delivery *in vivo*.
**Biolistics**: delivery of genetic material to cells or organisms using a gene gun and (usually gold) nanoparticles.
**Bottleneck**: proposed means by which mitochondrial (mt)DNA content/heteroplasmy of the oocyte is defined by replication of only a subpopulation of mtDNA molecules in primordial germ cells.
**Copy number**: the total quantity of mtDNA molecules within a given cell.
**Guide RNA (gRNA)**: an RNA that is required for sequence specific manipulation of DNA by Cas9.
**Heavy strand**: the G-rich strand of mtDNA, termed heavy strand owing to its relative buoyancy on cesium chloride gradients.
**Heteroplasmy**: a phenomenon occurring when multiple mtDNA haplotypes coexist within a single cell or organism, for example a mutated mtDNA and a wild-type mtDNA, as in many mitochondrial DNA diseases.
**Inner mitochondrial membrane (IMM)**: the location of the respiratory chain and translocase/carrier proteins.
**Light strand**: the C-rich strand of mtDNA, termed light strand owing to its relative buoyancy on cesium chloride gradients.
**Mitochondrial replacement therapy**: an *in vitr*o fertilization (IVF)-based approach to prevent mitochondrial diseases in which the nuclear genetic content of an oocyte bearing a pathogenic mtDNA mutation is transplanted into an enucleated oocyte from a non-carrier, which, after fertilization, results in an almost complete purge of mutant mtDNA from resulting embryos, but produces offspring with DNA from three genetic sources: father, mother, and mitochondrial donor mother.
**Mitochondrial RNase P (mtRNAse P)**: a protein-only trimeric protein complex.
**Mitochondrial targeting sequence (MTS)**: canonically the MTS is an N-terminal amphipathic helix.
**PiIT N terminus (PIN)**: nuclease domain.
**Polynucleotide phosphorylase (PNPase)**: an RNA exonuclease/polymerase.
**Proteinaceous RNase P (PRORP)**: the name for both the subclass of RNase P that does not require RNA for catalysis and the catalytic protein subunit contained within PRORPs (also known as MRPP3).
**H1 RNA**: nuclear RNase P, catalytic RNA subunit.
**7-2 RNA**: RNase MRP, catalytic RNA subunit.
**5S rRNA**: 5S ribosomal RNA, a structural rRNA that is present in many systems.
**RNase MRP**: mitochondrial RNA-processing endonuclease, now thought to function in nuclear rRNA processing.
